# Is the lower atmosphere a readily accessible reservoir of culturable, antimicrobial compound-producing Actinomycetales?

**DOI:** 10.3389/fmicb.2015.00802

**Published:** 2015-08-04

**Authors:** Carolyn F. Weber, Jason T. Werth

**Affiliations:** Department of Biological Sciences, Idaho State University, PocatelloID, USA

**Keywords:** aerobiology, Actinomycetales, antibiotics, bioprospecting, atmosphere

## Abstract

Recent metagenomic studies have revealed that microbial diversity in the atmosphere rivals that of surface environments. This indicates that the atmosphere may be worth bioprospecting in for novel microorganisms, especially those selected for by harsh atmospheric conditions. This is interesting in light of the antibiotic resistance crisis and renewed interests in bioprospecting for members of the Actinomycetales, which harbor novel secondary metabolite-producing pathways and produce spores that make them well suited for atmospheric travel. The latter leads to the hypothesis that the atmosphere may be a promising environment in which to search for novel Actinomycetales. Although ubiquitous in soils, where bioprospecting efforts for Actinomycetales have been and are largely still focused, we present novel data indicating that culturable members of this taxonomic order are 3–5.6 times more abundant in air samples collected at 1.5, 4.5, 7.5, and 18 m above the ground, than in the underlying soil. These results support the hypothesis that mining the vast and readily accessible lower atmosphere for novel Actinomycetales in the search for undescribed secondary metabolites could prove fruitful.

## Introduction

Although the field of aerobiology predates Louis Pasteur’s classic experiments in the late 19th century, the atmosphere has recently emerged as one of the last great frontiers in the field of microbiology. Recent research has demonstrated that airborne microbes are more diverse than previously thought, with richness estimates of microbes in the atmosphere rivaling those of soil (Smith et al., 2013). Although a significant amount of effort in the field of aerobiology remains focused on airborne pathogens and the spread of infectious diseases in an increasingly global society, the new found diversity has at least a few researchers looking toward the atmosphere as a place to bioprospect for novel, beneficial microbes that may fuel biotechnological innovations (Polymenakou, 2012). The wide ranging and fluctuating temperatures, high levels of solar irradiation as well as the strong oxidizing and desiccating conditions in the atmosphere may select for novel microorganisms with unique metabolic properties (Polymenakou, 2012).

Such novel microorganisms may include bacteria in the order Actinomycetales. Numerous studies have documented that Gram positive, spore-forming bacteria, such as members of the Actinomycetales, dominate culture-dependent surveys of the atmosphere (Womack et al., 2010). Additionally, recent metagenomic studies have confirmed that Actinobacteria, which include Actinomycetales, are a ubiquitous component of the overall microbial composition in the atmosphere (Tringe et al., 2008; Bowers et al., 2011; Smith et al., 2013). At present, Actinomycetales, primarily within the genus *Streptomyces*, produce over 50% of the world’s clinically useful antibiotics (Liu et al., 2013). This, in combination with continued discovery of secondary metabolite-producing pathways in Actinomycetales genomes, maintains this taxon as a focal point in bioprospecting efforts. The World Health Organization’s declaring antibiotic resistance an international crisis (World Health Organization [WHO], 2001) has renewed interest in bioprospecting for novel antimicrobial compound-producing organisms to potentially fuel drug development pipelines.

However, to date, no one has considered the atmosphere as a potential place to mine for novel antibiotic-producing Actinomycetales. Many bioprospecting campaigns remain focused on soils (i.e., Barral et al., 2014). Athough Actinomycetales have a cosmopolitan distribution, efforts to cultivate novel members of this taxon have explored unique soils (e.g., Okoro et al., 2009) and extreme environments (e.g., sea ice; Romanenko et al., 2008). Efforts to enrich and select for Actinomycetales in culture, especially from soils and plant materials, have involved laborious procedures such as drying or heating samples to kill faster growing opportunistic bacteria (e.g., many Proteobacteria) that outcompete Actinomycetales in culture (Van Hop et al., 2011). In the context of recent discoveries of microbial diversity in the atmosphere and the known ecology of Actinomycetales, we propose the atmosphere as a vast and readily accessible reservoir that is worthy of mining for novel antibiotic-producing members of this taxonomic order. We report the results of a direct comparison of the culturable diversity of this bacterial order from soil and air, which indicates that novel Actinomycetales may be more readily cultured from air than from the underlying soils.

## Actinomycetales Ecology and Secondary Metabolite Production

Actinomycetales is an order within the phylum Actinobacteria. The order is aerobic, Gram positive and is known for its ability to produce mycelia that give it a fungal-like appearance when grown on agar plates (Goodfellow and Williams, 1983). Actinomycetales are commonly found in soils that are neutral to alkaline pH and well-drained, favoring their aerobic metabolism (Kämpfer, 2006). A few species within the *Streptomyces* are found in aquatic and marine environments, but many other genera within the Actinomycetales can be found in marine environments (Subramani and Aalbersberg, 2012).

The cosmopolitan distribution of Actinomycetales may be partly due to their ability to form spores, which can travel long distances in air (Hervas et al., 2009). A review by Womack et al. (2010) notes that Gram positive bacteria, including many that form spores, tend to dominate culture-dependent surveys of the atmosphere. DNA-based microbial surveys confirm that Actinomycetales are likely abundant in the atmosphere (Tringe et al., 2008; Bowers et al., 2011; Smith et al., 2013). For example, it has been found that Actinobacteria, which includes the Actinomycetales order, can comprise large percentages of sequences recovered from aerosols in Denver, Colorado (∼10–25%; Bowers et al., 2011), Singapore (5–20%; Tringe et al., 2008), and Transpacific plumes (5–15%; Smith et al., 2013).

*Streptomyces* is the best-known genus within the Actinomycetales order because of its discovered ability to produce secondary metabolites that have antibiotic properties; these secondary metabolites are produced in coordination with the complex morphological differentiation that involves the production of aerial hyphae and ultimately spores (Flärdh and Buttner, 2009). When conditions are favorable, a *Streptomyces* spore will produce germ tubes that grow into hyphae; hyphae grow via tip extension into the surrounding substrates (Flärdh and Buttner, 2009). When nutrients are depleted, secondary metabolite production and morphological differentiation is triggered, leading to the formation of aerial hyphae (Flärdh and Buttner, 2009). The complexity of secondary metabolite pathways, their role in microbial development as well as their ecological function is still a topic of research. Although we have come to know secondary metabolites, such as Streptomycin, as antibiotics, we must remember that therapeutic levels of such metabolites are orders of magnitude higher than those found in nature (Davies, 2006). In nature, secondary metabolites may serve a diversity of functional roles including agents of symbiosis, agents of metal transport, sex hormones, differentiation effectors and inhibitors or stimulators of germination (Demain and Fang, 2000). Understanding the conditions under which secondary metabolites play these roles could prove fruitful in activating novel pathways and discovering new drugs. Many of the known bioactive secondary metabolites, 45% of which are produced by Actinomycetales (Berdy, 2005), have antibiotic and antitumor properties.

As the antibiotic resistance crisis intensifies and an increasing number of research efforts focus on discovering novel secondary metabolites, it has been proposed that focus might benefit from a shift away from the *Streptomyces* to other genera within the Actinomycetales, or non-streptomycete actinomycetes (Jose and Jebakumar, 2013). Certainly, this is an avenue worth exploring in order to improve the chances of discovering new compounds. However, we caution against abandoning the examination of *Streptomyces* altogether; the genomics revolution, when turned toward antibiotic-producing bacteria, has revealed stunning and completely uncharacterized diversity in secondary metabolite biosynthesis pathways, even within “well-characterized” *Streptomyces* (i.e., *Streptomyces coelicolor*; Bentley et al., 2002). Most strains carry the capacity to produce 20–30 potential small-molecule secondary metabolites, of which perhaps only 2–3 might be characterized in any given strain (Nett et al., 2009). For instance, the *Streptomyces* erythraeus genome sequence revealed at least 27 biosynthesis genes or gene clusters that are associated with the production of secondary metabolites of unknown chemical composition and structure (Nett et al., 2009). Novel gene clusters identified among members of the *Streptomyces* via genomic sequencing include those that are associated with the production of hybrid polyketide synthase non-ribosomal peptide synthetase salinosporamide products and modified enediyne sporolide polyketides among others (Nett et al., 2009). There is even considerable diversity in secondary metabolite production among strains of the same species; in a bioinformatic comparison of *Streptomyces albus* strains, 48 unique biosynthetic gene clusters were identified, with each strain harboring at least one strain-specific cluster, many of which have not been characterized (Seipke, 2015). This suggests a vast and untapped reservoir of potentially novel antibiotic biosynthetic pathways among the diversity of gene clusters that await the discovery of the signals that awaken their expression. Fortunately, a large recent body of work has shown that many such gene clusters can indeed be awakened using simple chemical or biological stimuli that can reveal otherwise cryptic antibiotic biosynthesis (reviewed by Yoon and Nodwell, 2014). As mentioned above, many of these compounds may serve a multitude of ecological roles and understanding the conditions under which they are produced in nature may be the key to identifying products of “cryptic” biosynthesis pathways.

It has been well established that soils are a primary habitat for Actinomycetales, including those that produce antibiotics. As such, many bioprospecting efforts remain focused in this environment (e.g., Small World Initiative; Barral et al., 2014). However, as noted by Hayakawa (2008), the taxon of interest is often outcompeted by other microbes when soil microbial assemblages are cultivated on agar. This necessitates designing enrichment methods and growth media that reduce competition from other microbes. Some strategies have involved using carbon sources, such as humic acids, that are fairly recalcitrant to degradation and cannot be easily utilized by faster growing competitors, but are accessible to Actinomycetales (Hayakawa, 2008). Other methods have involved laborious and aggressive pretreatment of soil samples including exposure to dry heat, phenol and sucrose-gradient centrifugation (Hayakawa, 2008). Bioprospecting efforts that have taken place in marine environments have been successful in identifying new compounds, including those that have antitumor activity, but are also fraught with logistical challenges. Firstly, the desired taxa are not as abundant in sea sediments as they are in terrestrial soils and sampling sediments from deep environments such as the Mariana Trench (Olano et al., 2009) is costly and difficult. Additionally, the unique cultivation requirements for marine Actinomycetales (i.e., high salt) can pose obstacles in creating the ideal culture conditions to propagate the desired taxa (Olano et al., 2009). Given the above, mining the atmosphere presents three primary advantages: (1) the atmosphere is easily accessed from any location, (2) faster-growing soil bacteria that may outcompete Actinomycetales on agar plates may be selected against by the harsh conditions of the atmosphere (e.g., desiccation, UV-radiation), thus reducing competition in culture, and (3) an air sample may provide the opportunity to capture a more representative sample of microbes from the underlying environment than terrestrial environments, which are characterized by high degrees of spatial heterogeneity.

## Bioprospecting for Actinomycetales in the Lower Atmosphere

As many bioprospecting efforts for Actinomycetales are still focused on soils, we performed a direct assessment of the potential of the atmosphere to yield culturable Actinomycetales relative to the underlying soil and present evidence from this assessment that the atmosphere may be a more promising place to bioprospect for this taxon.

In two air-sampling campaigns (Fall, 2013), bacteria were cultivated on King’s B medium (2009; Cold Spring Harbor) with 50 μg mL^-1^ cycloheximide (KBC) from 45 air samples (100 L volumes) collected using a SAS Super 100 (Bioscience International, Rockville, MD, USA) on the roof of a three-story building on the Idaho State University campus (Pocatello, ID, USA) as described by Weber and Werth (2015). We isolated and purified 28 morphologically unique isolates and sequenced their 16S rRNA genes as previously described (Weber and King, 2012). BLAST analysis revealed that the isolates were 99–100% similar to species of *Streptomyces, Lentzea*, and *Arthrobacter*, many of which are undescribed or uncultured, and 50% of them produced antimicrobial compounds (**Table 1**).

**Table 1:** Bacterial isolates obtained during air sampling campaigns in October and November 2013, with the nearest BLAST hits to their 16S rRNA gene sequences.

Isolate	Nearest BLAST hit	% Similarity	Inhibitory activity
111113air4	*Streptomyces* badius partial 16S rRNA gene, strain CB00830	99	F (2), GN (10)
KB 23	*Streptomyces* luteireticuli strain NRRL B-12435 16S ribosomal RNA gene, partial sequence	98	F (3)
KB 22	*Arthrobacter* sp. WPCB182, 16S ribosomal RNA gene, partial sequence	99	-
KB 27	*Arthrobacter* sp. JSM 2215039 16S ribosomal RNA gene, partial sequence	98	-
KB 20	Uncultured bacterium clone 10-472 16S ribosomal RNA gene, partial sequence	99	-
KB 39	*Lentzea* sp. 173316 16S ribosomal RNA gene, partial sequence	98	-
LB 1	*Nocardiopsis* sp. TFS 91 16S rRNA gene, partial sequence	99	-
KB 24	*Streptomyces* alni strain D65 16S ribosomal RNA	99	-
111013air4	*Streptomyces europaeiscabiei* strain 08-46-04-2 (#50) 16S rRNA gene, partial sequence	99	F (5)
KB 36	*Streptomyces* sp. CAI-67, 16S ribosomal RNA gene, partial sequence	100	F (1)
KB 38	*Streptomyces* sp. CAI-67, 16S ribosomal RNA gene, partial sequence	99	-
KB 43	*Streptomyces* sp. WPCB180 16S ribosomal RNA gene, partial sequence	99	GN (2), F (2)
LB 4	*Streptomyces* sp. 13-2-25, 16S ribosomal RNA gene, partial sequence	99	-
111513air2	*Streptomyces* sp. 3490 16S rRNA gene, partial sequence	99	-
KB 29	*Streptomyces atrovirens*, partial 16S rRNA gene. Strain SW22	99	-
111513air5	*Streptomyces* sp. S42 16S ribosomal RNA gene, partial sequence	99	GP (7)
110913air1	*Streptomyces* sp. NEAU 16S ribosomal RNA gene, partial sequence	99	F (2)
111013air3	*Streptomyces* sp. NEAU-WS1 16S ribosomal RNA gene, partial sequence	99	GP (1)
KB 44	*Streptomyces* sp. O10 partial 16S rRNA gene, strain O10	99	-
KB 37	*Streptomyces* sp. N4-145 16S ribosomal RNA gene, partial sequence	100	GN (1)
111513air1	*Streptomyces* sp. P4(2014) 16S ribosomal RNA gene, partial sequence	99	GP (10)
KB 40	*Streptomyces* sp. ADI93-O2 16S ribosomal RNA gene, partial sequence	99	-
111013air2	*Streptomyces* sp. SXY49 16S ribosomal RNA gene, partial sequence	99	F (2)
KB 33	*Streptomyces* sp. TW2 16S ribosomal RNA gene, partial sequence	99	F (1)
KB 13	*Streptomyces* sp. DR7-13 16S ribosomal RNA gene, partial sequence	99	-
KB 35	*Streptomyces* sp. enrichment culture clone Y3087, 16S ribosomal RNA gene, partial sequence	99	-
KB 31	*Streptomyces succinus* strain IHB B 6872 16S ribosomal RNA gene, partial	99	-
KB 30	Uncultured *Streptomyces* sp. clone ASC836 16S ribosomal RNA gene, partial sequence	99	F (1)

*Inhibition assays were carried out on King’s B agar medium against a Gram negative bacterium (*Escherichia coli* K12), Gram positive bacterium (*Staphylococcus aureus*) and a fungus (*Alternaria alternata*); isolates were spotted onto King’s B agar medium and allowed to grow at room temperature for 14 days prior to being overlain with molten agar inoculated with *Escherichia coli* K12 or *Staphylococcus aureus* at an optical density (600 nm) of 0.32–0.36 or inoculated with *Alternaria alternata* at the edge of the plate. Assays were observed for zones of clearing, indicating the presence of an inhibitory substance, over the next 2 weeks. Cases where zones of clearing were observed are denoted with “GN” (Gram negative), “GP” (Gram positive) and “F” fungi; cases where no zones of clearing were observed are denoted “-”. The number in parentheses in the column labeled “inhibitory activity” is the size of the zone of clearing, which is the distance (mm) between the isolate and test species; potency of inhibitory substance may not be directly proportional to the size of the zone of clearing due to variable biomass of test isolate and unknown quantities of inhibitory metabolites produced among assays.*

The apparent ease with which Actinomycetales were cultured from air samples led us to examine the relative culturability of Actinomycetales from air and from soil, where many bioprospecting efforts for antibiotic-producing taxa have been focused (Tiwari and Gupta, 2013). In June 2014, we collected air by elevating a SAS Super 180 (Bioscience International, Rockville, MD, USA) with a pulley system attached to an 18 m tower at the ISU Challenge Course (Pocatello, ID, USA). At 1.5, 4.5, 7.5, and 18 m above the ground, 180 L of air was sampled onto each of five KBC plates that had been overlaid with autoclaved polycarbonate membranes. Five surface soil samples (0–2 cm) were aseptically collected near the base of the tower. Bacteria from each soil sample (2.9–6.3 g) were extracted in 25 mL of 1X phosphate buffer; 100 μL of each extract was spread plated on top of a KBC agar plate overlaid with an autoclaved polycarbonate membrane. After incubating all 25 plates at room conditions for 2 weeks, membranes were peeled from the agar, capturing the total culturable biomass for DNA extraction. The 16S rRNA gene was PCR amplified from each DNA extract according to the methods of Caporaso et al. (2012) and sequenced on the Illumina MiSeq at the ISU Molecular Research Core Facility (Pocatello, ID, USA). Prior to taxonomic classification, sequences were quality checked and trimmed using mothur software (Schloss et al., 2009). Sequences with homopolymers >7 bases, average quality scores <25, ambiguous bases or that were chimeras were eliminated from the dataset. Sequences were taxonomically classified using the classify.seqs command in mothur and the Silva bacteria database and taxonomy as the reference. Only classifications with confidence levels of ≥80 were considered. After trimming and removing poor quality sequences, an average of 162,738 sequences remained in each sequence library (Supplementary Table S1). All libraries have been deposited into MG-RAST (http://metagenomics.anl.gov) with the following ID numbers: 4603809.3, 4603785.3, 4603786.3, 4603787.3, 4603788.3, 4603789.3, 4603790.3, 4603791.3, 4603792.3, 4603793.3, 4603794.3, 4603795.3, 4603796.3, 4603797.3, 4603798.3, 4603799.3, 4603800.3, 4603801.3, 4603802.3, 4603803.3, 4603804.3, 4603805.3, 4603806.3, 4603807.3, 4603808.3.

**FIGURE 1:**
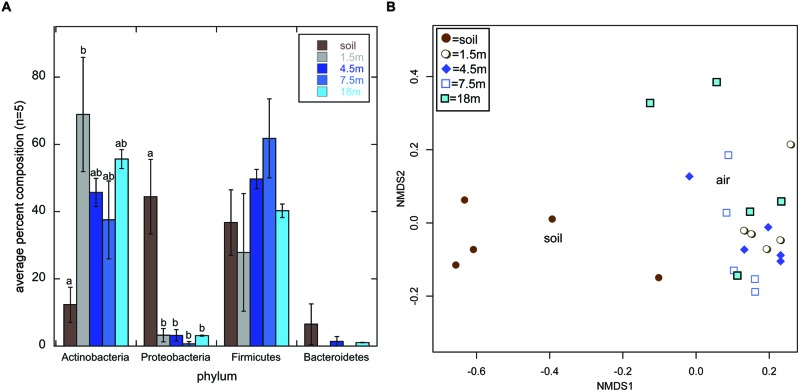
**Average percent composition (±1SE; *n* = 5) of each library type (soil or air collected at 1.5, 4.5, 7.5, or 18 m above the ground) based on the phylum-level classification of sequences using the classify.seqs command in mothur (Schloss et al., 2009) and the Silva Bacteria Database (confidence threshold = 80) (A).** Small letters denote statistical significance based on a Welch’s ANOVA of the arcsine square root transformation of the percentages followed by pairwise-*t*-tests with a Bonferroni Correction for multiple comparisons (α = 0.05). Absence of small letters indicates lack of statistical significance. **(B)** Displays a multidimensional scaling (mds) plot generated using the Bray–Curtis distance metric to calculate distances between all 25 sequence libraries based on the genus-level classification data of sequences within the Actinomycetales order.

Actinobacteria was the most frequently detected phylum across all libraries (44.8% of all sequences); however, they comprised only 12.3% of soil sequence libraries, while they comprised 37.5–68.9% of air sequence libraries (**Figure 1A**). The opposite trend was observed for Proteobacteria, which comprised a statistically higher percentage of sequences in soil libraries than in air libraries (**Figure 1A**). KBC is routinely used to select for *Pseudomonas* sp. (e.g., Johnsen and Nielsen, 2006); indeed, this was the most frequently detected genus within the Proteobacteria soil sequences, but 380 was the highest number of *Pseudomonas* sequences detected in any air library (Supplemental Information S1). This suggests that commonly used, complex growth media, such as KBC, is suitable for the growth of Actinobacteria, but they may get outcompeted in cultures when fast growing opportunistic genera (i.e., *Pseudomonas*), are also present in environmental samples even at relatively low abundance; 16S rDNA sequencing of soils near the sampling site indicate that the abundance of *Pseudomonas* may be rather low, comprising 0.03% of 205,711 total sequences obtained (Weber, 2015).

Actinomycetales comprised 99.9% of all Actinobacteria, but their genus-level composition differed dramatically between the soil and air samples (**Figure 1B**). On average, *Streptomyces* comprised 51.6% of sequences in soil libraries, but <2.6% of any of the air sequence libraries. This suggests that bioprospecting efforts targeting non-*Streptomyces* Actinomycetales in air may have more promise than those focused on soil. This is interesting in light of recent proposals that non-*Streptomyces* Actinomycetales may nourish drug development pipelines (Jose and Jebakumar, 2013). Additionally, Actinomycetales sequences that were unclassified at the genus level, potentially representing novel taxa, comprised 18.7–25.4% of the air sequence libraries, on average, but only 8.5% of the soil sequence libraries.

## Outlook

The lower atmosphere contains bacteria that originate from soils, but it appears that sampling air may provide a selective advantage for culturing Actinomycetales with reduced competition from faster growing, opportunistic taxa. The potential to cultivate non-*Streptomyces*, Actinomycetales is particularly interesting, but our demonstration that undescribed, antimicrobial compound-producing *Streptomyces* can also be easily cultivated from air is valuable as well, as the complete array of antimicrobial compounds that *Streptomyces* may be able to produce is not known. Genomic studies indicate that a typical strain of *Streptomyces* can produce 20–30 secondary metabolites, but relatively few have been characterized (Nett et al., 2009). This may be due to our relatively scanty knowledge of the ecological roles of these compounds in nature and the conditions under which they are synthesized (Davies, 2006). Therefore, our collective results lead us to propose the lower atmosphere as a vast and readily accessible environment in which bioprospecting for antimicrobial compound-producing microbes could prove fruitful. Further assessment of the potential for bioprospecting in the lower atmosphere to yield novel Actinomycetales should sample air onto a greater variety of growth media at different geographical locations during different seasons to determine how generalizable the trends in our findings might be.

## Conflict of Interest Statement

The authors declare that the research was conducted in the absence of any commercial or financial relationships that could be construed as a potential conflict of interest.

## Supplementary Material

TABLE S1 **Taxonomic classification of sequences in all libraries**.
